# Copolyesters Based on 2,5-Furandicarboxylic Acid (FDCA): Effect of 2,2,4,4-Tetramethyl-1,3-Cyclobutanediol Units on Their Properties

**DOI:** 10.3390/polym9090305

**Published:** 2017-08-24

**Authors:** Jinggang Wang, Xiaoqing Liu, Jin Zhu, Yanhua Jiang

**Affiliations:** 1 Ningbo Institute of Materials Technology and Engineering, Chinese Academy of Sciences, Ningbo 315201, China; wangjg@nimte.ac.cn (J.W.); jzhu@nimte.ac.cn (J.Z.); jyhua@nimte.ac.cn (Y.J.); 2 University of Chinese Academy of Sciences, Beijing 100049, China

**Keywords:** 2,5-Furandicarboxylic acid (FDCA), poly (ethylene 2,5-furandicarboxylate) (PEF), poly(propylene 2,5-furandicarboxylate) (PPF), poly(butylene 2,5-furandicarboxylate) (PBF), 2,2,4,4-tetramethyl-1,3-cyclobutanediol (CBDO)

## Abstract

Bio-based polyesters derived from 2,5-furandicarboxylic acid (FDCA), including poly (ethylene 2,5-furandicarboxylate) (PEF), poly(propylene 2,5-furandicarboxylate) (PPF), and poly(butylene 2,5-furandicarboxylate) (PBF) have been synthesized and modified with 2,2,4,4-tetramethyl-1,3-cyclobutanediol (CBDO). Copolyesters with increased glass transition temperature, good barrier and better mechanical properties, as well as higher transparency were reported in this work. The chemical structures, composition, and sequence distribution of the copolyesters were determined by ^1^H NMR and ^13^C NMR. The degree of random (*R*) was close to 1 for all the copolyesters, indicating their random chemical structures. With the introduction of 10% CBDO units, the semi-crystalline PEF and PPF were changed into completely amorphous polyesters and the higher transparency was easily achieved. The glass transition temperature was increased from 87 °C for PEF to 91.1 °C for PETF-18, from 55.5 °C for PPF to 63.5 °C for PPTF-18, and from 39.0 °C for PBF to 43.5 °C for PBTF-18. The barrier properties investigation demonstrated that although the O_2_ and CO_2_ barrier of PEF/PPF/PBF were decreased by the addition of CBDO units, the modified copolyesters still showed good barrier properties.

## 1. Introduction

In recent years, due to diminishing crude oil reserves and worsening environmental pollution, more and more attention has been paid to the synthesis of polymers derived from renewable resources to replace the petroleum-based ones. However, their thermal or mechanical properties are still subject to improvement when compared with the petroleum-based engineering plastics like poly(ethylene terephthalate) (PET) and polycarbonate (PC) [1,2,3]. The lack of aromatic or rigid segments in their molecular architecture should be responsible for the relatively low performance. Therefore, exploring the new bio-based platform chemicals with unique structures and then developing polymeric materials with a higher performance–price ratio is always a matter of importance.

2,5-furandicarboxylic acid (FDCA) is exactly the promising bio-based platform chemical, which can be derived from cellulose or hemicellulose. It has been selected as one of the top 12 value-added chemicals derived from biomass by the US Department of Energy. It is also referred to as a “sleeping giant” by DuPont and DSM due to its great potential as a bio-based substitute for terephthalate (TPA) [4]. In fact, as early as 1940s, the FDCA-based polymers had been firstly reported in Celanese’s work. However, due to the difficulty of obtaining FDCA with high purity and its unacceptable price, hardly no work about polymers derived from FDCA has been reported since. Nowadays, with the rapid development of biotechnology and chemical industry, the large scale industrialized production of FDCA is being realized. Therefore, more and more polymeric materials—Such as polyesters, [5,6,7,8,9] polyamides, [10,11,12] polyurethanes [13,14], and epoxy resins [15]—Have been synthesized from FDCA rather than TPA. Among them, the FDCA-based polyesters—Including poly (ethylene 2,5-furandicarboxylate) (PEF) [8,16], poly(propylene 2,5-furandicarboxylate) (PPF) [17], and poly(butylene 2,5-furandicarboxylate) (PBF) [18,19,20,21]—Might be the most popular stars, which demonstrates similar mechanical and thermal properties as well as better barrier properties when compared with the petroleum-based counterparts (PET/PPT/PBT) [22,23]. As we know, the total world production of PET, PPT, and PBT was more than 50 Mt per year, if they could be replaced by the bio-based PEF/PPF/PBF or their copolyesters, great progress towards the sustainable development of polymer industry could be achieved. Therefore, the investigation of the synthesis and modification of PEF, PPF, and PBF has become a hot topic in both the industrial and academic communities.

Up to now, synthesis strategies and basic thermal-physic properties of PEF/PPF/PBF have been widely investigated [5,6,7,8,16,17,18,19,20,21]. However, being fresh members in the family of polyesters, their properties are still subject to improvement in order to meet the various demands from industry [24,25]. For example, the crystallization behaviors of PEF/PPF/PBF were well studied [17,18,26]. Results demonstrated that they were all semi-crystalline polyesters with medium high glass transition temperature. In addition, their crystallizabilities were much poorer when compared with those of PET/PPT/PBT, which would severely limit their fields of application, especially when the highly transparent and heat-resistant properties were required. Up to now, a large quantity of chemical compounds—including isosorbide and isoidide [27], 1,4-cyclohexanedimethanol (CHDM) [28], terephthalic acid [29] and different aliphatic diols [27,30]—Have been tried to modify PEF/PPF/PBF. For example, Gomes et al. [27] synthesized several kinds of FDCA-based copolyesters from isosorbide and isoidide. Results indicated that by using these cyclic diols, amorphous polymers with higher *T*_g_ could be obtained. However, their reported molecular weight was too low to be useful. 1,4-cyclohexanedimethanol (CHDM) was also employed to modify PEF and results showed that after the introduction of CHDM, the toughness of PEF could be significantly increased without sacrificing its *T*_g_ and barrier properties in a great degree. Unfortunately, these copolyesters were still semi-crystalline polymers and high transparency was difficult to achieve [28]. In one word, completely amorphous FDCA-based copolyesters with increased *T*_g_, higher transparency as well as good barrier properties and mechanical properties are still targets to be achieved, especially when they are used as packaging materials.

To the best of our knowledge, selecting an ideal cyclic compound as the co-monomer is a well-recognized method to strengthen polyesters without sacrificing their thermal properties. 2,2,4,4-tetramethyl-1,3-cyclobutanediol (CBDO) is one of the unique cyclic aliphatic diols which has been tried as a replacement of bisphenol-A (BPA) to enhance the properties of several polyesters. For instance, Eastman Chemical Company has developed several kinds of CBDO-containing copolyesters, including Tritan^TM^, and demonstrated that the introduction of CBDO would lead to higher *T*_g_, excellent hydrolytic stabilities, and good toughness. Recently, Beall [31] synthesized an amorphous CBDO-based terephthalate copolyester, which showed much higher *T*_g_ and good toughness. Zhang [32] prepared a series of multiblock copolyesters from high-*T*_g_ polyester precursors containing CBDO segments. Inspired by the previous publications, in this work, CBDO was employed to modify the FDCA-based polyesters including PEF, PPF, as well as PBF, and a series of copolyesters, such as poly(ethylene-*co*-2,2,4,4-tetramethyl-1,3-cyclobutanediol 2,5-furandicarboxylate) (PETF), poly(propylene-*co*-2,2,4,4-tetramethyl-1,3-cyclobutanediol 2,5-furandicarboxylate) (PPTF), and poly(tetramethylene-*co*-2,2,4,4-tetramethyl-1,3-cyclobutanediol 2,5-furandicarboxylate) (PBTF) with high molecular weight were synthesized. Their chemical structures and compositions were determined by ^1^H NMR and ^13^C NMR. The crystallization behaviors, mechanical properties, and optical and barrier properties of the copolyesters were investigated by DSC, TGA, tensile testing, UV–visible spectrophotometer and gas permeability tester. The objective of this paper was to provide us FDCA-based copolyesters with increased *T*_g_ and good mechanical properties as well as better transparency without sacrificing their barrier properties severely. Based on which, the bio-based substitute for PET, PPT, and PBT with better comprehensive performance could be developed and used as packaging materials.

## 2. Materials and Methods

### 2.1. Materials

CBDO with the purity of 99% was purchased from Suzhou Yacoo Chemical Reagent Co., Ltd. (Suzhou, China). Ethanediol (EG, 99%), propanediol (PPD, 99%), and butanediol (BDO, 99%) were purchased from Aladdin Reagent Co. Ltd. (Shanghai, China). Antimony trioxide (III, 99.99%), zinc acetate (99%), trifluoroacetic acid, phenol, and tetrachloroethane were obtained from Sinopharm Chemical Reagent Co., Ltd. (Shanghai, China). Bio-based 2,5-furandicarboxlic acid (99.9%) was purchased from Chemtarget Technologies Co., Ltd. (Mianyang, China). It is the oxidation product of 5-hydroxymethylfurfural (HMF), which is the important intermediate from fructose to FDCA. All the chemicals were used as received without any purification.

### 2.2. Synthesis of Dimethyl Furan-2,5-dicarboxylate (DMFD)

The synthesis of DMFD followed the procedures described in previous literature [28]. Its chemical structure was confirmed by ^1^H NMR and ^13^C NMR before the polycondensation.

^1^H NMR (400 MHz, DMSO-d_6_, δ, ppm): 7.41 (2H), 3.88 (6H).

^13^C NMR (400 MHz, DMSO-d_6_, δ, ppm): 119.6, 146.7 (4C), 158.7 (2C), 52.9 (2C).

### 2.3. Synthesis of PEF/PPF/PBF and Their Copolyesters

The two-stage melt polycondensation reaction was taken to prepare PEF, PPF, PBF, PETF, PPTF, and PBTF from diol (EG, PPD, BDO), CBDO, and DMFD with the molar ratio of diol/DMFD was 1.6. All the reactions were conducted in a 500 mL three-necked round bottom flask equipped with a mechanical stirrer with torque indicator. The predetermined diol (EG, PPD, or BDO), CBDO, DMFD (0.50 mol) and catalyst (Zinc acetate, 0.2 mol % based on DMFD) were added into the flask. Then the reaction was conducted at 180 °C for 4 h under the protection of nitrogen. After almost all the theoretically produced methanol was distilled out, subsequently the second portion of catalyst was added (antimony trioxide (III), 0.15 mol·% based on DMFD). After that, the reaction temperature was increased to 230–245 °C and pressure of the reaction system was gradually reduced to 10–20 Pa for a certain time until the torque value of the stirrer reached a fixed value to ensure similar viscosity of all the products. Finally, the reaction system was returned to the normal atmospheric pressure by the introduction of N_2_ and the target product was obtained.

The synthetic condition and composition of different polyesters were listed in Table 1. The different samples were named as PETF-10, PPTF-10, PBTF-10, PETF-18, PPTF-18, and PBTF-18, where the numbers 10 and 18 represent the mole percentage of CBDO in the copolyesters. For example, PPTF-10 stands for the sample in which the mole ratio of CBDO/(CBDO + PPD) in the copolyesters was about 0.1.

### 2.4. Measurements

The viscosity of synthesized copolyesters was measured by Ubbelohde viscometer in a 25 °C water bath. The diameter of the capillary tube was 0.792 mm. The phenol and tetrachloroethane mixture (1/1, *w/w*) was used as the solvent with the concentration of copolyester was 0.125 g/25 mL. The equation [η] = η_sp_/*c* was used to calculate the intrinsic viscosity [η], where η_sp_ = (*t*_1_ − *t*_0_)/*t*_0_, *t*_0_ is the elution time of solvent and *t*_1_ the elution time of polyesters solutions [5].

Chemical structures, compositions, and sequence distribution of the synthesized copolyesters were determined by ^1^H NMR and ^13^C NMR in CF_3_COOD using a Bruker AVIII400 NMR spectrometer (Karlsruhe, Germany) at 25 °C. Tetramethysilane (TMS) was used as the internal standard. DSC measurements were performed on a differential scanning calorimeter (Mettler-Toledo DSC I, Zurich, Switzerland) under the nitrogen flow rate of 50 mL/min. Before the measurement, it was calibrated with pure indium and zinc standards to ensure accuracy. A sample of about 5–6 mg was placed in an alumina pan before it was heated to 250 °C with the heating rate of 10 °C/min. All the samples should be maintained at this temperature for 3 min to erase thermal history. Then it was cooled down to room temperature at a rate of 10 °C/min and heated to 250 °C with the same heating rate for the second time. All the DSC curves were recorded for thermal properties analysis. Thermal stability evaluation was conducted using a TGA instrument (Mettler-Toledo TGA/DSC thermogravimetric analysis, Zurich, Switzerland). For each measurement, 6–10 mg sample was placed in a ceramic furnace and the TGA curves under nitrogen atmosphere were recorded from 50 to 800 °C for thermal stability analysis. The X-ray diffraction (XRD) patterns were collected on a Bruker D8 ADVANCE, using Cu-Kα radiation (40 kv, 40 mA) with a scanning range from 5° to 50°. The scanning speed was 0.2°/s. Ultraviolet–visible (UV–vis) spectra of the polyester films were recorded on a Lambda 950 UV/Vis/NIR spectrophotometer (Waltham, MA, USA). Tensile testing was performed on an Instron 5567 tensile machine (Norwood, MA, USA) with a 500 N load cell. The crosshead speed was 10 mm/min. The dumbbell-shaped samples were prepared by hot press-molding at 230–240 °C with the dimension of 20.0 mm in length, 2.0 mm in neck width and 1.0 mm in thickness. Before testing, all the samples were conditioned at 25 °C for over 48 h. For accuracy, at least five specimens were tested for each sample.

The barrier properties were measured by Labthink VAC-V2 gas permeability tester (Jinan, China). The experiment was conducted at 30 °C with the relative humidity (RH) of 50%. The oxygen and carbon dioxide with the purity of 99.99% were used in our experiment. The completely amorphous polyesters films were prepared following the melt-press/quench procedures. The round films with the diameter of 97 mm and the surface area of 38.5 cm^2^ were used and the testing range is 0.05–50,000 cm^3^/m^2^·24 h·0.1 MPa.

## 3. Results and Discussion

### 3.1. Synthesis of FDCA-Based Copolyesters

**Scheme 1:**
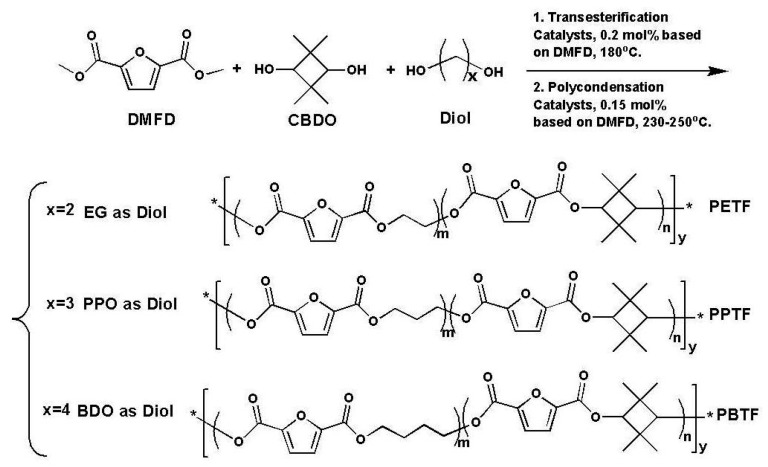
Synthesis and chemical structures of different copolyesters.

The FDCA-based polyesters (PEF, PPF, PBF) and copolyesters (PETF-10, PPTF-10, PBTF-10, PETF-18, PPTF-18, and PBTF-18) were synthesized following a two-step procedure consisting of transesterification and polycondensation shown in Scheme 1. In order to avoid the thermal degradation of FDCA during the synthetic process, FDCA is usually converted into DMFD by the esterification with methanol at first (Supporting Information Figures S1 and S2) and then the transesterification is conducted. For the transesterification reaction, 0.2 mol·% zinc acetate (based on the amount of DMFD) was used as the catalyst and the temperature was set at 175–180 °C. In 3–4 h, after all the theoretical methanol was distilled out of the reaction system, 0.15 mol·% of antimony trioxide (III) (based on the amount of DMFD) was added and the following polycondensation reaction was conducted at 230–245 °C under the pressure of 10–20 Pa. During the reaction, the torque of the stirrer was increased slowly with the viscosity growth of polymers. It finally got up to 270–320 N·cm in another 3–4 h for all the samples. The intrinsic viscosity of the products, PEF/PPF/PBF and the copolyesters—PETF-10, PETF-18, PPTF-10, PPTF-18, PBTF-10, and PBTF-18—were determined to be 0.74–0.98 dL/g (Table 1). In addition, their molecule weight was calculated based on the equation [η] = 4.68 × 10^−4^·*M*_v_^0.68^ using Mark–Houwink parameters and the obtained data has been listed in Table 1. Obviously, the *M*_v_ of PEF was as high as 69,000.

### 3.2. Chemical Structures and Composition of the Synthesized Copolyesters

**Figure 1:**
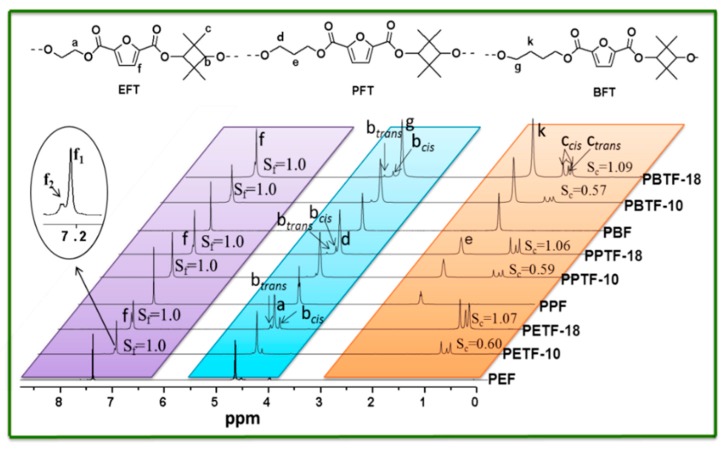
The ^1^H NMR spectra of PEF, PPF, PBF, and the copolyesters modified with CBDO.

The chemical structures of FDCA-based polyesters and copolyesters were determined by ^1^H-NMR and ^13^C NMR. Figure 1 shows the ^1^H NMR spectra and characteristic peak assignment of PEF, PPF, PBF, PETFs, PPTFs, and PBTFs (Supporting Information Tables S1–S3). Obviously, as for PEF, the chemical shift of CH in the furan ring (f_1_) and CH_2_ in ethylene glycol (a) appeared at 7.20 ppm and 4.63 ppm, respectively. As for PPF, the characteristic peaks showing at 4.43 and 2.15 ppm were assigned to CH_2_ in propylene glycol unit (d, e). The signals at 4.61 ppm (g) and 2.08 ppm (k) were associated with CH_2_ in butanediol unit for PBF. For the characteristic peak assignments of PETF-10, PPTF-10, PBTF-10, PETF-18, PPTF-18, and PBTF-18, the chemical shift of CH_2_ in diol unit was retained, and the signals for CH in the furan ring were varied between 7.20–7.23 ppm (f1) and 7.24–7.26 ppm (f_2_) due to the influence of different ester bonds connecting with the furan rings. As we know, CBDO is a non-planar structure. The *cis*-form and *trans*-form will demonstrate different characteristic chemical shifts in the ^1^H NMR spectra. In Figure 1, the chemical shift for CH (marked with b_cis_) in *cis*-form CBDO was shown at 4.53 ppm and the peak for CH (marked with b_trans_) in *trans*-form CBDO was appeared at 4.70 ppm. In addition, the single peak showing at 1.14 ppm was assigned to CH_3_ (c_t__rans_) in the *trans*-form CBDO due to the equivalent chemical situation. While the signals for CH_3_ (c_cis_) in the *cis*-form CBDO were split into multiple peaks showing at 1.07 ppm and 1.24 ppm [33]. Accordingly, the content of CBDO in the copolyesters’ chain could be determined by the intensity of peak c to that of peak f. The integration of each peak was shown in Figure 1 and the composition of copolyesters was listed in Table 1. The mole percentage of CBDO in the copolyesters was a little higher than the feed ratio of CBDO to diol. The reason might be that the CBDO was more difficult to be distilled out due to its higher boiling point and bulk structure when compared with ethanediol, propanediol, and butanediol, which was similar to the previous results [34].

**Figure 2:**
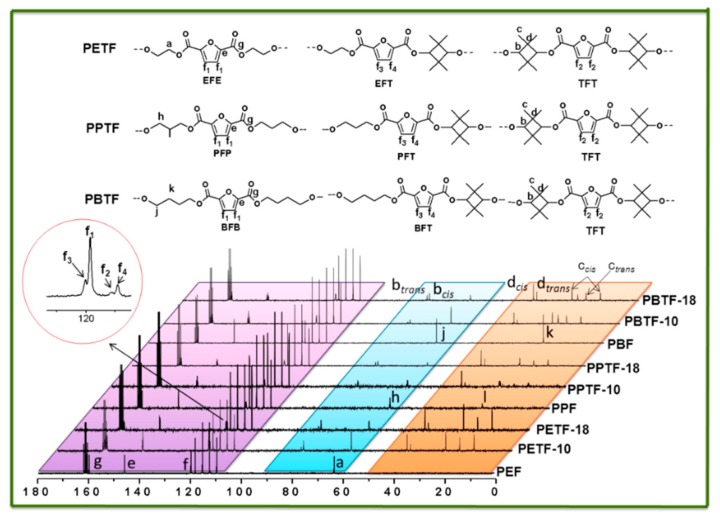
The ^13^C NMR spectra of PEF, PPF, PBF, and the copolyesters modified with CBDO.

In Figure 2, the ^13^C NMR spectra of PEF, PPF, PBF and the copolyesters modified with CBDO were shown. It is a fact that the chemical shifts of aromatic carbons in the furan ring will be influenced by the collecting ester bonds. Based on which, the sequence distribution of synthesized copolyesters could be determined accordingly. For analysis convenience and easy expression, PETF-18 was taken as an example here. In Figure 2 (^13^C NMR spectrum for PETF-18), the secondary carbon atom in both EFE and TFT units showed only one peak (peak f_1_ and peak f_2_). The different peaks in PETF-18 corresponded to the different types of carbon atoms f_1_, f_2_, f_3_, and f_4_, which presented different polyester chain (Supporting Information, Figure S3). The peak intensity I_f1_, I_f2_, I_f3_, and I_f4_ corresponded to the molar percent of EFE, EFT, TFE, and TFT unit. Then the Equations (1)–(6) were employed to calculate the number average sequence lengths of units *n*_n,EF_, *n*_n,TF_ and the degree of random *R*. Using the same method, the sequence distribution analysis of other copolyesters were also conducted and the calculated results were listed in Table 1. For easy understanding, in Table 1, the capital “D” stood for different diols (ethanediol, propanediol or butanediol), “T” stood for CBDO, and “F” represented FDCA. It was easy to notice that the degree of random (*R*) for PETF-10, PPTF-10, PBTF-10, PETF-18, PPTF-18, and PBTF-18 were all about 1, which indicated their random copolymer characteristics.

*N*_EFE_ = *I*_f1_/(*I*_f1_ + *I*_f2_ + *I*_f3_ + *I*_f4_) (1)

*N*_EFT+TFE_ = (*I*_f3_ + *I*_f4_)/(*I*_f1_ + *I*_f2_ + *I*_f3_ + *I*_f4_) (2)

*N*_TFT_ = *I*_f2_/(*I*_f1_ + *I*_f2_ + *I*_f3_ + *I*_f4_) (3)

*n*_n,EF_ = 1 + 2*I*_f1_/(*I*_f3_ + *I*_f4_) (4)

*n*_n,TF_ = 1 + 2*I*_f2_/(*I*_f3_ + *I*_f4_) (5)

*R* = 1/*n*_n,EF_ + 1/*n*_n,TF_ (6)

### 3.3. Thermal Properties Investigation

**Figure 3:**
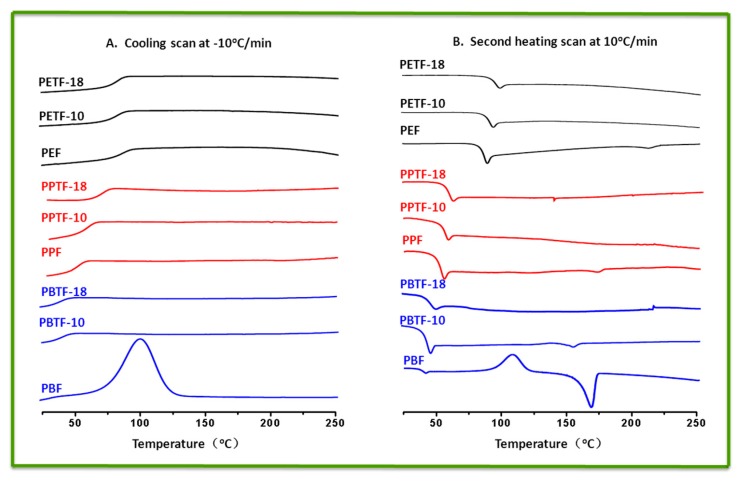
DSC curves for PEF, PPF, PBF, and the copolyesters modified with CBDO.

The thermal properties of PEF/PPF/PBF and their copolyesters were investigated by DSC. The recorded cooling and heating curves were shown in Figure 3 and the corresponding data was summarized in Table 2. Figure 3A was the cooling scan from 250 °C to room temperature for all the samples and only PBF displayed a crystallization peak at 109.4 °C. In Figure 3B, PEF, PPF, and PBF showed the melting peak at 211.9, 173.6, and 168.8 °C (*T*_m_) with the melting enthalpy (Δ*H*_m_) of 0.9, 0.5, and 31.1 J/g, respectively. For the copolyesters, when the content of CBDO reached 10%, only a small melting peak at 153.4 °C was observed for PBTF-10. When the content of CBDO was increased to 18%, both PETF-18 and PPTF-18 as well as PBTF-18 did not show any melting peaks except for the glass transition signals. These results indicated that the crystallizability of PBF was much better than that of PEF and PPF. With the introduction of CBDO, the crystallizability of the copolyesters was significantly decreased and PETF-18, PPTF-18, as well as PBTF-18 might be completely amorphous. It was easy to notice that all the copolyesters showed only one *T*_g_, which was in accordance with their random structures determined by ^13^C NMR. In addition, the *T*_g_ was increased obviously after the introduction of CBDO, from 87 °C for PEF to 91.1 °C for PETF-18, from 55.5 °C for PPF to 63.5 °C for PPTF-18 and from 39.0 °C for PBF to 43.5 °C for PBTF-18. The rigidity of CBDO might be responsible for the increased *T*_g_.

**Figure 4:**
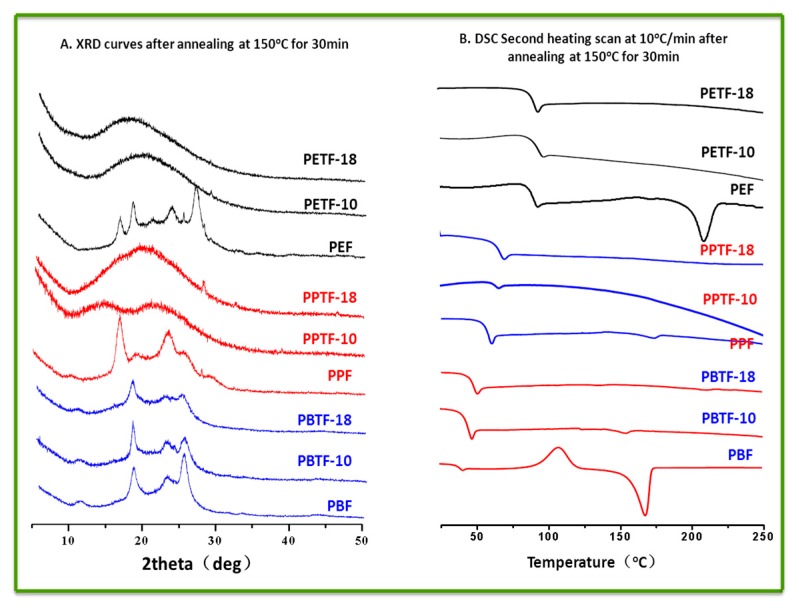
XRD and DSC curves for PEF, PPF, PBF, and their copolyesters after being annealed at 150 °C for 30 min.

In order to further investigate the crystallization behaviors of the synthesized copolyesters, the wide angel X-ray diffraction was conducted for the samples after being annealed at 150 °C for 30 min. In Figure 4, PEF exhibited strong reflections at 2θ values of 16.2°, 18.1°, 23.4°, 26.7°, and a lower intensity diffraction peak at 20.8°, which represented the PEF crystallization [34]. As for PPF and PBF, they also exhibited strong reflections at the 2θ values of 19.1°, 23.2°, 18.1°, and 25.1°, respectively. However, for the copolyesters, except for the annealed PBTF-10 and PBTF-18, no obvious reflection peaks were noticed, which indicated the amorphous characteristic of PETF-10, PETF-18, PPTF-10, and PPTF-18 even after annealing at 150 °C. Figure 4B shows the DSC curves of PEF/PPF/PBF and their copolyesters after annealing treatment. Besides PETF-10, PETF-18, PPTF-10, and PPTF-18, the PBTF-18 also did not show obvious melting peak. However, based on the weak XRD reflection peaks of PBTF-18 shown in Figure 4A, the trace crystallization of PBTF-18 was formed during the annealing treatment and its melting enthalpy might be too low to be monitored by DSC during the heating process, which was different from the completely amorphous PETF-10, PETF-18, PPTF-10, and PPTF-18.

The thermal stabilities of PEF, PPF, PBF, and their copolyesters were investigated by TGA (Supporting Figure S4). The key parameters for thermal stability evaluation, such as the 5% weight loss temperature (*T*_5%_), the temperature at which the maximum decomposition rate was observed (*T*_d,max_) and the residual mass at 650 °C (*R*_650_), were all listed in Table 2. Obviously, PEF, PPF, and PBF showed similar thermal stabilities and they were not affected by the introduction of CBDO units. They demonstrated similar *T*_5%_ and *T*_d,max_, ranging from 365 to 370 °C and from 397 to 405 °C, respectively. The residual masses at 650 °C for PEF, PETF-10, and PETF-18 were a little higher than those of PPF, PBF, and their copolyesters (PPTF-10, PPTF-18, PBTF-10, and PBTF-18). The reason might be that the weight ratio of aromatic furan ring unit was higher in the PEF systems than in PPF and PBF systems.

### 3.4. Mechanical Properties

The tensile properties of synthesized copolyesters were measured by tensile testing. Their tensile modulus, tensile strength, and elongation at break are shown in Table 3. Obviously, PEF shows a tensile modulus of 2800 MPa and a tensile strength of 85 MPa, much higher than the literature values 2450 and 35 MPa, respectively [24]. The reason might be that the molecular weight of PEF in this work was much higher. The low elongation at break of 5% was similar to the values in other publications (2.8% in [23] and 4.2% in [5]). For PBF, it has a tensile modulus of 2000 MPa, a strength of 62 MPa, and the elongation at break of 290%, which were similar to the previously reported values. When the CBDO unit was incorporated into PEF, PPF, and PBF, the tensile modulus and strength of the copolyesters were all increased accordingly. The tensile modulus and tensile strength of PETF-18 were 3300 and 95 MPa, respectively, indicating an increment of 17.8% in modulus and 11.7% in strength compared with those of PEF. As for PPTF-18 and PBTF-18, the tensile modulus and tensile strength were also increased. Especially for the tensile strength, it was increased from 54MPa for PPF to 78 MPa for PPFT-18 (increased 44%) and from 62 MPa for PBF to 80 MPa for PBTF-18 (increased 29%). However, the elongation at break of PETF-18 and PBTF-18 were all decreased a little compared with those of PEF and PBF. The elongation at break of PPTF-18 was decreased much lower than that of PPF. The reason for the modulus and strength increment along with the decreasing elongation might be the more rigid molecular chain after the introduction of CBDO.

### 3.5. Transparency Investigation

**Figure 5:**
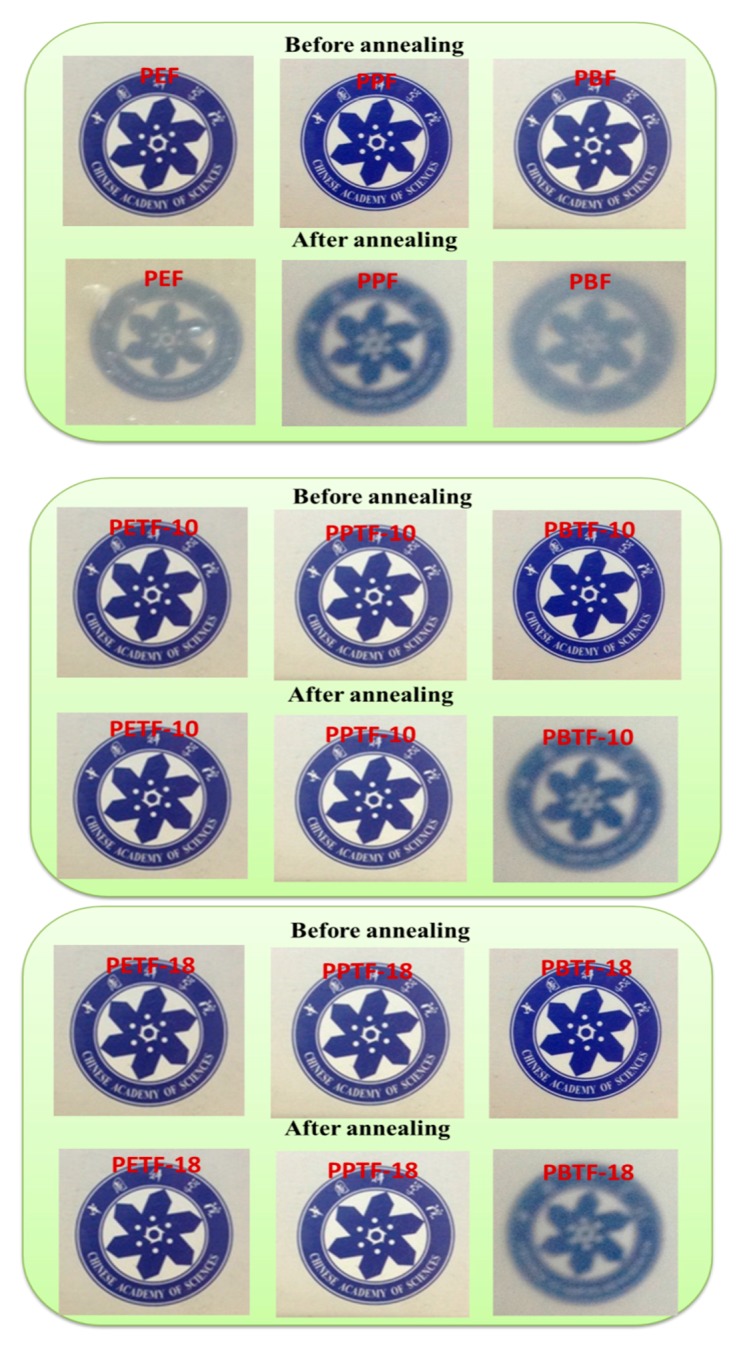
Optical image of PEF, PPF, PBF, and the copolyesters modified with CBDO films before and after annealing at 150 °C for 30 min.

**Figure 6:**
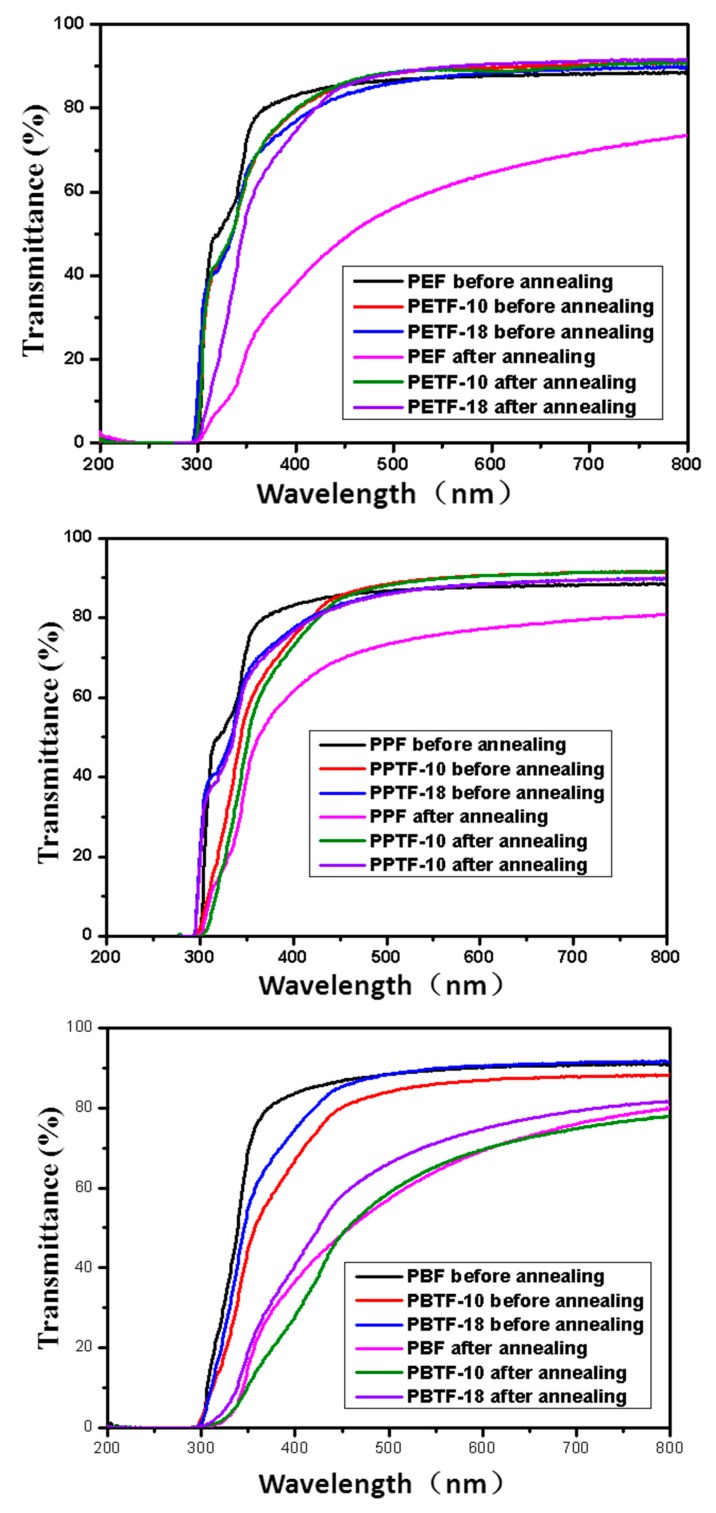
UV–visible spectra of PEF, PPF, PBF, and copolyesters modified with CBDO before and after annealing at 150 °C for 30 min.

For packaging materials, sometimes transparency is the key factor in determining their application fields. Figure 5 shows the optical images for amorphous PEF, PPF, PBF, and their copolyesters films prepared by melt-press/quench procedures before and after annealing at 150 °C for 30 min. Obviously, all the films were clear and transparent before annealing at 150 °C for 30 min. The images in Figure 5 also indicated that following the synthesis procedures described in the Experimental Section, the white color PEF/PPF/PBF and their coployesters could be prepared. After being annealed, the transparent PEF, PPF, and PBF films became opaque. As for the copolyesters, except PBTF-10 and PBTF-18, the appearance of PETF-10, PETF-18, PPTF-10, and PPTF-18 films did not show any change due to their completely amorphous characteristics even after annealing treatment. In order to quantitatively investigate their optical properties, the UV–visible spectra for all the films were shown in Figure 6 and the transmittance data was listed in Table 4. After annealing at 150 °C for 30 min, the transmittance of PEF film was decreased from 88.4% to 71.9% by cutoff at 700 nm and from 85.7% to 48.9% by cutoff at 450 nm. While the transmittance of PPF film was decreased from 88.4% to 80.0% by cutoff at 700 nm and from 85.7% to 69.5% by cutoff at 450 nm. As for the PBF film, its transmittance was also decreased from 90.8% to 75.9% by cutoff at 700 nm and from 86.7% to 48.2% by cutoff at 450 nm. However, the transmittance of PETF-10, PETF-18, PPTF-10, and PPTF-18 films was remained almost unchanged before and after isothermal treatment at 150 °C. This result was in line with the above conclusion based on thermal properties investigation. Therefore, it could be concluded that, after the introduction of the CBDO segment, the higher transparency was easier to achieve.

### 3.6. Barrier Properties

The CO_2_ and O_2_ barrier properties of PEF, PPF, PBF, and their copolyesters (PETF-10, PETF-18, PPTF-10, PPTF-18, PBTF-10, PBTF-18) were investigated according to the method described in the Measurement Section. In Table 5 and Table 6, the as-obtained results were collected. For easy comparison, the well-known polyester with good gas barrier properties, poly(ethylene naphthalate) (PEN), was taken as the control. In addition, the popular Barrier Improvement Factor (BIFP), which represents the permeability coefficient of CO_2_ or O_2_ in PET divided by the CO_2_ or O_2_ permeability coefficient in other materials (PEF, PPF, PBF, PEN, et al.), was also employed in Table 5 and Table 6 [22,23,35,36]. It is easy to understand that the higher the BIFP, the better the barrier properties. From Table 5, the CO_2_ BIFp for PEF, PPF, and PBF were 13.0, 8.1, and 7.2, respectively. This indicated that the PEF, PPF, and PBF have better CO_2_ barrier property than PEN. The reason might be corresponding to the increased CO_2_ solubility in PEF, PPF, and PBF, which originated from the greater interaction between CO_2_ and the polar furan moiety and then reduced the diffusivity of CO_2_. After the introduction of CBDO, the CO_2_ permeability coefficient of all the copolyesters were increased accordingly. It was increased from 0.010 for PEF to 0.059 for PETF-18 with the BIFP of 2.2, from 0.016 for PPF to 0.020 for PPTF-18 with the BIFp of 6.5 and from 0.018 for PBF to 0.055 for PBTF-18 with the BIFp of 2.4. Although, the introduction of CBDO lowered the barrier property to CO_2_ of PEF, PPF, and PBF, the copolyesters containing 10% CBDO units still showed better CO_2_ barrier properties when compared with that of PEN (CO_2_ BIFp of PEN is 2.9 reported in reference [35]).

In Table 6, evidenced by the O_2_ BIFp of 5.5 for PEF, 4.0 for PPF and 3.3 for PBF, they (PEF, PPF, and PBF) all exhibited better O_2_ barrier properties compared with PEN. While for PETF-18, PPTF-18, and PBTF-18, the O_2_ BIFp was lowered, indicating increased O_2_ permeation of copolyesters after the incorporation of CBDO. Nevertheless, PETF-10 and PPTF-18 still showed better O_2_ barrier properties relative to PEN based on the O_2_ BIF_P_ listed in Table 6.

## 4. Conclusions

FDCA-based polyesters—including PEF, PPF, as well as PBF—And their copolyesters modified with CBDO were synthesized successfully. All the copolyesters showed the degree of random close to 1, indicating their random structures. The introduction of CBDO units led to the increased glass transition temperature, tensile modulus, and strength as well as decreased crystallizability. When the content of CBDO reached 10%, the semi-crystalline PEF and PPF were became completely amorphous (PETF-10, PETF-18, PPTF-10, and PPTF-18) and then high transparency was easily achieved. Although the barrier properties to O_2_ and CO_2_ of PEF, PPF, and PBF were all decreased with the introduction of CBDO, the modified copolyesters containing 18% CBDO still showed better barrier properties to CO_2_ when compared with PEN, and the ones containing 10% CBDO demonstrated better barrier properties to O_2_. This paper provided us FDCA-based copolyesters with increased *T_g_*, better mechanical properties, and higher transparency as well as good barrier properties. Based on which, the bio-based substitutes for PET, PPT, and PBT with better performance could be developed, especially when used as packaging materials.

## Figures and Tables

**Table 1:** Composition and sequence distribution of the synthesized copolyesters.

Sample	In Feed		In Copolyesters ^a^		Triads Component ^b^ mol %		Average Sequence Length		Degree of Random	Intrinsic Viscosity		Molecule Weight
	DMFD (mol)	CBDO (mol)	Diol (mol)	CBDO/Diol (mol)	*N*_DFD_	*N*_DFT+TFD_	*N*_TFT_	*N*_DF_	*N*_TF_	*R*	[η] ^c^	*M*_v_^d^
PEF	0.50	0	0.8	0:100	100	-	-	-	-	-	0.92	6.9 × 10^4^
PETF-10	0.50	0.075	0.725	10.3:89.7	78.1	20.1	1.8	8.7	1.1	0.97	0.79	5.6 × 10^4^
PETF-18	0.50	0.12	0.68	18.2:81.8	69.0	27.4	3.6	5.0	1.3	0.99	0.74	5.1 × 10^4^
PPF	0.50	0	0.80	0:100	100	-	-	-	-	-	0.88	6.5 × 10^4^
PPTF-10	0.50	0.075	0.725	9.8:90.2	79.4	18.7	1.9	9.5	1.2	0.94	0.93	7.1 × 10^4^
PPTF-18	0.50	0.12	0.68	17.8:82.2	67.2	27.9	4.9	5.8	1.3	0.94	0.76	5.3 × 10^4^
PBF	0.50	0	0.80	0:100	100	-	-	-	-	-	0.98	7.6 × 10^4^
PBTF-10	0.50	0.075	0.725	9.6:90.4	80.2	17.9	1.9	9.9	1.2	0.93	0.96	7.4 × 10^4^
PBTF-18	0.50	0.12	0.68	17.9:82.1	69.3	26.1	4.6	5.9	1.3	0.94	0.92	6.9 × 10^4^

^a^ mole fraction of CBDO in the polyesters, determined by integration of ^1^H NMR spectra. ^b^ mole fraction of DFD, DFT + TFD, and TFT units calculated on the basis of ^13^C NMR analysis, in which “D” stands for diols (ethanediol, propanediol, or butanediol), “T” stands for CBDO, and “F” represents FDCA. ^c^ intrinsic viscosity obtained with an Ubbelohde viscometer. ^d^ molecule weight determined by equation [η] = 4.68 × 10^−4^·*M*_v_^0.68^ using Mark–Houwink parameters.

**Table 2:** Thermal properties of PEF, PPF, PBF, and the copolyesters.

Sample	DSC							TGA		
	Second Heating Scan at 10 °C/min					Scan after Annealing at 150 °C for 30 min				
	*T*_g_ (°C)	*T*_cc_ (°C)	Δ*H*_cc_ (J/g)	*T*_m_ (°C)	Δ*H*_m_ (J/g)	*T*_m_ (°C)	Δ*H*_m_ (J/g)	*T*_d,5%_ (°C)	*T*_d,max_ (°C)	*R*_650_ (wt %)
PEF	87.0	nd	nd	211.9	0.9	208.2	29.6	365	401	7.9
PETF-10	90.9	nd	nd	nd	nd	nd	nd	368	403	7.4
PETF-18	91.1	nd	nd	nd	nd	nd	nd	369	400	7.6
PPF	55.5	nd	nd	173.6	0.5	173.6	1.7	367	405	6.1
PPTF-10	61.1	nd	nd	nd	nd	nd	nd	370	403	6.5
PPTF-18	63.5	nd	nd	nd	nd	nd	nd	361	397	5.5
PBF	39.0	109.4	30.4	168.8	31.1	168.6	32.6	367	398	5.8
PBTF-10	42.5	nd	nd	154.4	0.5	153.4	0.8	368	405	4.6
PBTF-18	43.5	nd	nd	nd	nd	nd	nd	365	404	4.8

**Table 3:** Tensile properties of PEF, PPF, PBF, and the copolyesters modified with CBDO.

Sample	Tensile Modulus (MPa)	Tensile Strength (MPa)	Elongation at Break (%)
PEF	2800 ± 120	85 ± 9	5 ± 1
PETF-10	3100 ± 100	97 ± 4	9 ± 5
PETF-18	3300 ± 100	98 ± 2	4 ± 1
PPF	2700 ± 30	53 ± 2	50 ± 7
PPTF-10	2750 ± 20	63 ± 3	56 ± 11
PPTF-18	2800 ± 40	78 ± 11	30 ± 10
PBF	2000 ± 30	62 ± 3	290 ± 6
PBTF-10	2100 ± 80	72 ± 2	274 ± 10
PBTF-18	2200 ± 60	80 ± 2	220 ± 18

**Table 4:** Transmittance of PEF, PPF, PBF, and the copolyesters modified by CBDO at different wavelength (the deviation is about ±1% for all the reported data here).

Sample	Amorphous		150 °C for 30 min	
	Transmittance % (450 nm)	Transmittance % (700 nm)	Transmittance % 450 nm)	Transmittance % (700 nm)
PEF	85.7	88.4	48.9	71.9
PETF-10	85.1	90.8	86.0	90.4
PETF-18	83.9	90.2	84.3	89.8
PPF	85.7	88.4	69.5	80.0
PPTF-10	85.5	91.1	84.6	90.2
PPTF-18	84.4	89.7	83.7	89.7
PBF	85.7	90.8	48.2	75.9
PBTF-10	84.1	89.8	48.4	74.8
PBTF-18	85.3	91.3	60.1	79.0

**Table 5:** CO_2_ barrier properties of PEF, PPF, PBF, and the copolyesters modified with CBDO.

Sample ^a^	Temperature (°C)	CO_2_ Permeability Coefficient (Barrer ^b^)	BIF_P_	Reference
PET	30	0.13	1	This work
PEF	30	0.010	13.0	This work
PETF-10	30	0.019	6.8	This work
PETF-18	30	0.059	2.2	This work
PPF	30	0.016	8.1	This work
PPTF-10	30	0.018	7.2	This work
PPTF-18	30	0.020	6.5	This work
PBF	30	0.018	7.2	This work
PBTF-10	30	0.027	4.9	This work
PBTF-18	30	0.055	2.4	This work
PET	30	0.32	1	[35]
PEN	30	0.11	2.9	[35]

^a^ The test performed at low pressure (≤0.1001 MPa); ^b^ 1 barrer = 10^−10^ cm^3^·cm/cm^2^·s·cm Hg.

**Table 6:** O_2_ barrier properties of PEF, PPF, PBF, and the copolyesters modified with CBDO.

Sample ^a^	Temperature (°C)	O_2_ Permeability Coefficient (Barrier ^b^)	BIF_P_	Reference
PET	30	0.060	1	This work
PEF	30	0.011	5.5	This work
PETF-10	30	0.013	4.6	This work
PETF-18	30	0.028	2.1	This work
PPF	30	0.09	6.7	This work
PPTF-10	30	0.010	6.0	This work
PPTF-18	30	0.036	1.7	This work
PBF	30	0.018	3.3	This work
PBTF-10	30	0.025	2.4	This work
PBTF-18	30	0.042	1.4	This work
PET	30	0.054	1	[34]
PEN	30	0.019	2.9	[34]

^a^ The test performed at low pressure (≤0.1001 MPa); ^b^ 1 barrier = 10^−10^ cm^3^·cm/cm^2^·s·cm Hg.

## Supplementary Materials

The following are available online at www.mdpi.com/2073-4360/9/9/305/s1, Figure S1: ^1^H NMR spectra of dimethyl furan-2,5-dicarboxylate; Figure S2: ^13^C NMR spectra of dimethyl furan-2,5-dicarboxylate; Figure S3: Chemical strucutres of EFE, EFT, TFT and the peak assignment in ^13^C NMR spectra; Figure S4: TGA for PEF, PPF, PBF, PETF-10, PPTF-10, PBTF-10, PETF-18, PPTF-18, PPTF-18, and PBTF-18. Table S1: The ^1^H NMR signal assignments for PEF and PETF 10/18; Table S2: The ^1^H NMR signal assignments for PPF and PPTF 10/18; Table S3: The ^1^H NMR signal assignment for PBF and PBTF 10/18.
