# Supplementary material for: Copolyesters Based on 2,5-Furandicarboxylic Acid (FDCA): Effect of 2,2,4,4-Tetramethyl-1,3-Cyclobutanediol Units on Their Properties
Source: Polymers (Basel). 2017 Aug 24;9(9):305. doi: 10.3390/polym9090305 (PMC6418770; doi:10.3390/polym9090305)
Supplement: Supplementary file 1 [file polymers-09-00305-s001.pdf]

Supporting Information

Copolyesters Based on 2,5-Furandicarboxylic acid (FDCA): Effect of 2,2,4,4-Tetramethyl-1,3-Cyclobutanediol Units on Their Properties

Jinggang Wang [1,2] , Xiaoqing Liu [1]*, Jin Zhu [1] and Yanhua Jiang [1]

[1] Ningbo Institute of Materials Technology and Engineering, Chinese Academy of Sciences, Ningbo, Zhejiang 315201, (P. R. China)

[2] University of Chinese Academy of Sciences, Beijing 100049 (P. R. China)

*Correspondence: liuxq@nimte.ac.cn (Xiaoqing Liu); Tel.: 86-574-86685925

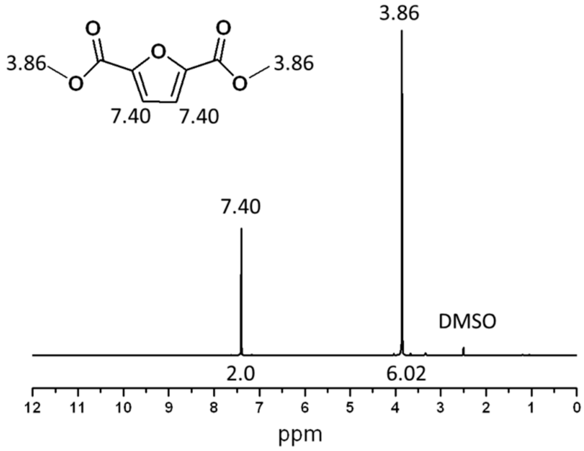

Figure S1 ^{1}H-NMR spectra of dimethyl furan-2,5-dicarboxylate

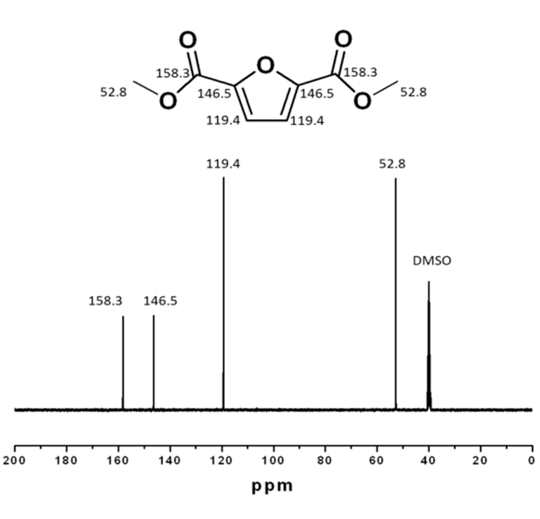

Figure S2 ^{13}C-NMR spectra of dimethyl furan-2,5-dicarboxylate

Table S1 The ^{1}H-NMR signal assignments for PEF and PETF 10/18

Sample	f_1	f_2	a	b_{cis}	b_{trans}	c_{cis}	c_{trans}
	CH in furan ring	CH in furan ring	CH_2 in EG	CH in cis-CBDO	CH in trans-CBDO	CH_3 in cis-CBDO	CH_3 in trans-CBDO
PEF (ppm)	7.20	-	4.63	-	-	-	-
PETF10/18	7.20-7.23	7.24-7.26	4.63	4.53	4.70	1.07,1.24	1.14

Table S2 The ^{1}H-NMR signal assignments for PPF and PPTF 10/18

Sample	f_1	f_2	d	e	b_{cis}	b_{trans}	c_{cis}	c_{trans}
	CH in furan ring	CH in furan ring	CH_2 in PPD	CH_2 in PPD	CH in cis-CBDO	CH in trans-CBDO	CH_3 in cis-CBDO	CH_3 in trans-CBDO
PPF	7.20	-	4.43	2.15	-	-	-	-
PPTF10-18	7.20-7.23	7.24-7.26	4.43	2.15	4.53	4.70	1.07,1.24	1.14

Table S3 The ^{1}H-NMR signal assignment for PBF and PBTF 10/18

Sample	f_1	f_2	g	k	b_{cis}	b_{trans}	c_{cis}	c_{trans}
	CH in furan ring	CH in furan ring	CH_2 in BDO	CH_2 in BDO	CH in cis-CBDO	CH in trans-CBDO	CH_3 in cis-CBDO	CH_3 in trans-CBDO
PBF	7.20	-	4.61	2.08	-	-	-	-
PBTF10-18	7.20-7.23	7.24-7.26	4.61	2.08	4.53	4.70	1.07,1.24	1.14

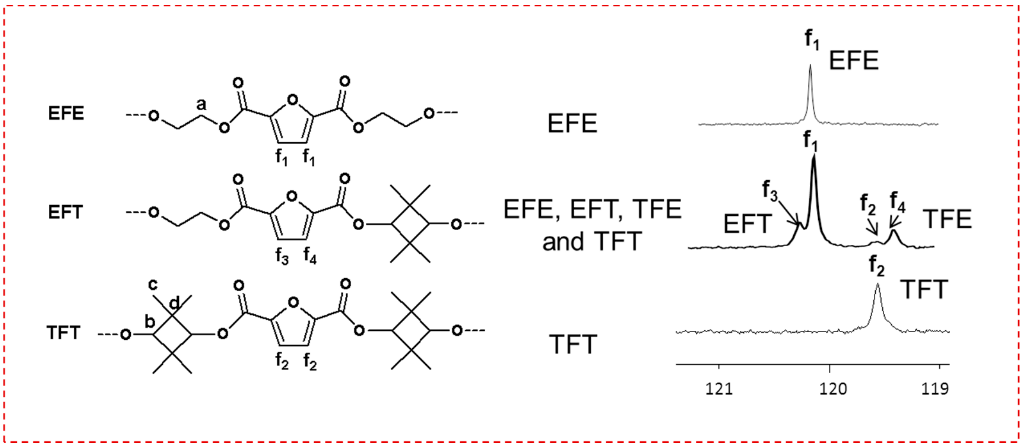

Figure S3 Chemical structures of EFE, EFT, TFT and the peak assignment in ^{13}C-NMR spectra

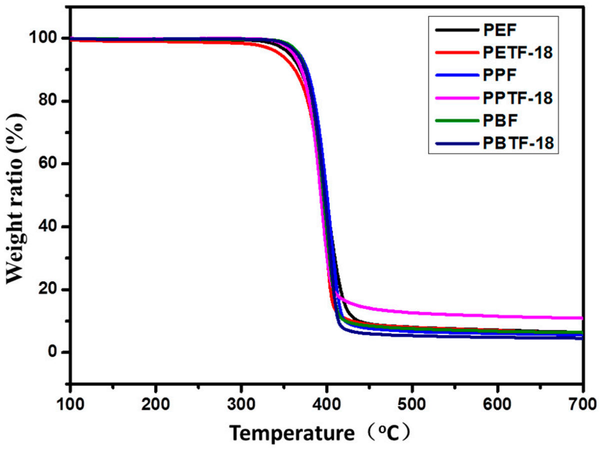

Figure S4 TGA curves for PEF, PPF, PBF, PETF-18, PPTF-18 and PBTF-18
